# The safety and efficacy of sequential intramuscular/oral ziprasidone treatment of acute episode in patients with schizophrenia: a multicenter, open-labeled study

**DOI:** 10.1186/s12888-023-04588-0

**Published:** 2023-03-15

**Authors:** Yaxue Wu, Yanli Li, Weiye Liang, Luyuan Bai, Jianjin Yu, Keqing Li, Yunshu Zhang, Yanmei Guo, Zenglong Liu, Jian Wang, Congpei Zhang, Xijin Wang, Jia Xu, Liping Liu, Juan Li, Fude Yang

**Affiliations:** 1 Peking University HuiLongGuan Clinical Medical School, Beijing Huilongguan Hospital, Beijing, People’s Republic of China; 2 The Mental Health Center of Hebei Province, Baoding, People’s Republic of China; 3 The Sixth People’s Hospital of Hebei Province, Baoding, People’s Republic of China; 4 Harbin First Specialized Hospital, Harbin, People’s Republic of China; 5 Beijing Geriatric Hospital, Beijing, People’s Republic of China

**Keywords:** Ziprasidone, Sequential therapy, Schizophrenia, Agitation

## Abstract

**Background:**

Ziprasidone mesylate injection is an atypical antipsychotic drug which is recently approved in China. In combination with its oral formulation, sequential therapy with ziprasidone brings new interventions to patients with agitation in the acute phase of schizophrenia. The purpose of this 7-day multicenter study conducted in China was to evaluate the efficacy and safety of ziprasidone sequential treatment through intramuscular/oral routes in agitated patients with schizophrenia.

**Methods:**

A total of 95 patients were enrolled from three centers in this study. The study duration was 7 days. In the first 3 days, subjects were administered an intramuscular injection of ziprasidone 10–40 mg daily and started sequentially with oral ziprasidone 40–80 mg at dinner (or lunch) from the day of the last intramuscular injection. In the following 4 days, according to the severity of the symptoms and the drug response, 120–160 mg of ziprasidone was orally administered daily. In total, six visits were scheduled to assess the Positive and Negative Syndrome Scale (PANSS), the Behavioral Activity Rating Scale (BARS), the Clinical Global Impression of Severity (CGI-S), and Improvement (CGI-I) scores throughout the procedure. Lastly, adverse events were recorded during treatment.

**Results:**

Out of the 95 patients that were enrolled, 83 cases were effectively completed. Visits 3, 4, 6, PANSS, and PANSS-excited component (PANSS-EC) subscale points, and Visit 2–Visit 6 viewpoints, BARS scale points, and baseline scores denote a progressive downward trend (*P* < 0.001). In this study, 62 adverse events were reported. The most common adverse events were extrapyramidal symptoms (EPS) (23 cases) and excessive sedation(10 cases), and 13 cases of prolonged QTc interval were reported.

**Conclusions:**

Ziprasidone IM demonstrated significant and rapid reduction in agitation, and sequential oral formulation keep stability and continuation of the treatment can further ensure efficacy. Ziprasidone sequential therapy may provide a new approach to acute agitation in schizophrenic patients.

**Trial registration:**

The Chinese Clinical Trials Registry; URL: https://www.chictr.org.cn: ChiCTR-OIC-16007970.

## Background

Agitation symptoms are common among people with mental disorders and need to be addressed immediately to prevent adverse events that can put patients, staff, and others at risk [1]. The United States Food and Drug Administration Center for Drug Evaluation and Research defines agitation as an excessive motor activity associated with a feeling of inner tension [2]. The behavioral syndrome of cross-disease classification is characterized by increased speech or behavioral activity, irritability, and uncooperative and threatening attitudes, whereby partial agitation may result in aggressive sexual violence [1, 3]. Schizophrenia is a chronic disease, but agitation accompanied by destruction and violence is very common in its acute phase [4]. A past study indicated that as many as 2 million emergency department visits in the United States per year may involve agitated psychiatric patients, with schizophrenia as the underlying cause in 21% of these visits [5]. A multicenter survey of 14 hospitals in China, conducted in 2014, revealed that the prevalence of mental illness accounted for 47.5% of the total number of cases [6]. Despite the lack of data on the incidence of agitation in schizophrenia, according to a survey on aggressive behavior in hospitalized patients with schizophrenia, the incidence rate was between 9.1–49.6%, with an average of 28.0% [7]. Zhou et al. performed a meta-analysis of 19 studies comprising 3,941 schizophrenia patients in a Chinese psychiatric ward and showed a pooled prevalence of 35.4% (95% confidence interval [CI], 29.7% – 41.4%) [8]. At any time, the presence of agitation in subjects can endanger other patients or themselves and facilitate exacerbation. Therefore, timely treatment of agitation in the acute phase is integral to treating schizophrenia.

The goal of dealing with agitation is to ensure the safety of the personnel while helping patients control their emotions and maintain or regain control of their behavior while simultaneously avoiding the use of constraints and coercive measures to achieve these goals [9]. According to the recommendation of the best practices in evaluating and treating agitation (BETA), the complete management of agitation consists of classification and diagnosis, early utilization of interpersonal calming skills, and medical intervention [10, 11]. As early as the year 2000, relevant scholars have studied the pathophysiological mechanisms associated with agitation. Several potential pathophysiological abnormalities were found to be mediated by the dysregulation of dopaminergic, serotonergic, noradrenergic, and GABA-ergic systems [12]. The selection of drugs for controlled agitation is also based on this research. At present, for patients with schizophrenia, a medication commonly used in the management of agitation includes typical and atypical antipsychotics and benzodiazepines. Typical neuroleptic injections, such as haloperidol injections, have been frequently used to treat patients with severe agitation. Nevertheless, intramuscular (IM) first-generation antipsychotics cause extrapyramidal symptoms (EPS), such as akathisia and dystonia [13]. Benzodiazepines, such as diazepam, lorazepam, and midazolam, can be effectively controlled through IM injection or oral administration [14–16], but these medications may have the potential for excessive sedation, respiratory depression, or hypotension. Furthermore, benzodiazepines do not affect the patient’s psychiatric symptoms and simply serve as a “stopgap”. In recent years, an increasing number of atypical antipsychotics (i.e., aripiprazole, olanzapine, and ziprasidone) have been approved for the treatment of agitation in mental illness [15, 17, 18].

From the standpoint of safety and convenience, oral medications are considered preferable to parenteral administration. Nevertheless, when the patient’s agitation symptoms are too serious, they often cannot cooperate with the treatment. IM anti-psychotic drugs can not only treat patients but are also more convenient than oral medication in terms of calming the patients. In this case, we need to first administer an IM drug to control agitation in the acute phase of schizophrenia, and then, to improve the patient’s compliance, transit to an oral formulation of the same or a different atypical antipsychotic [19]. In particular, a method called sequential therapy has been globally used across various medical fields in the past, including antibiotics, cancer treatment, and the eradication of *Helicobacter pylori*, but has been scarcely applied in the treatment of mental illness, especially in China. This disparity is mainly due to the lack of IM dosage forms in novel Chinese antipsychotics. Further, classic antipsychotics, such as haloperidol, are less likely to be used as the first-line treatment because of apparent side effects. Currently, ziprasidone is the only antipsychotic that can provide oral and intramuscular formulations in Chinese mental hospitals.

Ziprasidone is a 5-hydroxytryptamine-2A (5-HT2A)/dopamine-2(D2) antagonist and has higher in vitro 5-HT2A/D2 receptor affinity than other first-line atypical antipsychotics. Ziprasidone also interacts effectively with 5-HT2C, 5-HT1D, and 5-HT1A receptors in the human brain tissue, and has a low affinity for alpha1-adrenergic receptors, histamine H1, and muscarinic M1 receptors [20]. In clinical trials, oral ziprasidone (80–160 mg/day) demonstrated rapid and effective management of the positive and negative symptoms of schizophrenia and is well tolerated [21]. Notably, the medication is well tolerated because of fewer extrapyramidal adverse reactions, no significant weight gain, and menstrual changes. Furthermore, it does not cause elevated serum prolactin, particularly in female and adolescent schizophrenic patients. It has been validated from A 6-week study by Brook et al. and a European study comparing ziprasidone with haloperidol in sequential IM/ oral treatment (a subanalysis of the ZIMO trial), compared with classic antipsychotic haloperidol injections, ziprasidone has superior effective, fewer side effects, and more socially cost-effective [13, 22].

Studies from China have also confirmed that with the use of intramuscular ziprasidone to control agitation symptoms in schizophrenia, the incidence of extrapyramidal syndrome is lower than with the use of haloperidol injection [23, 24].

Due to the pharmacological properties of ziprasidone and its clinical advantages, the IM/oral sequential therapy of ziprasidone is being clinically applied to control the agitation symptoms of the acute phase of schizophrenia. An open-label study conducted by Mautone et al. in 2011 confirmed the effectiveness of this approach [19]. Essentially, we would need to conduct similar research in more centers to further understand the characteristics of its clinical application. The purpose of this study was to investigate the efficacy and safety of sequential treatment with ziprasidone intramuscular/oral dosage forms in Chinese agitated patients. In this study, we hypothesized that ziprasidone sequential therapy can effectively and safely control agitation in patients with acute schizophrenia. We will make a detailed summary of the actual user experience of the drug, and compare whether there are any differences in efficacy and safety in patients from two different regions of China.

## Methods

### Study setting and design

This study represents an open-label, single-arm, multicenter, phase IV prospective trial that lasted for 7 days. The trial consisted of two phases: the screening/baseline phase and the ziprasidone treatment phase. Specifically, the trial was conducted in three centers in China and enrolled 95 subjects.

The ziprasidone injections and oral preparations utilized in the research were provided by Pfizer Pharmaceuticals. The project has been registered in the Chinese Clinical Trials Registry before recruiting subjects (Reg No. ChiCTR: ChiCTR-OIC-16007970, Reg time: 22/02/2016). All researchers participated in the GCP training.

In the screening/baseline phase (i.e., Visit 1), written informed consent from the subject and their legal guardian was obtained before study entry, and the inclusion and exclusion criteria were reviewed to verify the subject’s eligibility.

The treatment phase was divided into the IM and oral periods. In the IM period, ziprasidone IM (10–40 mg/d) was administered for up to 3 days. After the IM treatment, the initial dose of oral ziprasidone was in the range of 40–80 mg and could be ingested before dinner (or lunch) on the day of the last injection. Subjects then received a flexible dosage of oral ziprasidone, 120–160 mg/day, depending on the clinical response until day 7.

### Subjects

Adult (18 − 65 years old) patients diagnosed with schizophrenia according to the Diagnostic and Statistical Manual of Mental Disorders Fourth Edition (DSM-IV) definition were eligible for inclusion if they met the following criteria: (a) hospitalized at the screening phase and previously no-antipsychotic-use patients who could remain in the hospital during the study period; (b) acute exacerbation of schizophrenia with a score between 70 to 120 (total score:210) on the Positive and Negative Syndrome Scale (PANSS) [25] and could receive intramuscular medication according to the investigator’s judgment; (c) with a minimum score of 14 on the PANSS-excited component (PANSS-EC, comprised P4: excited, P7: Hostility, G4: Physical tension, G8: Uncooperative, G14: Impulse-control disorder) [26], and a score not less than four in at least one item.

The patient was excluded on the following conditions: (a) agitation due to delirium, seizures, affective psychosis, poisoning, or substance abuse withdrawal reactions; (b) relevant history or current presence of any cardiovascular, respiratory, neurological, renal, hepatic, endocrine, immunological, or other systemic diseases; (c) confirmed clinically significant abnormal laboratory values; (d) clinically significant electrocardiogram (ECG) abnormality; (e) subjects with a history of QTc prolongation or a pre-drug QTc of 450 ms or greater; (f) subjects reporting serum K + or Mg2 + levels beyond the normal range; (g) history of malignant syndrome or tardive dyskinesia history; (h) pregnant or lactating women; (i) concomitant use of drugs that may induce QTc prolongation during the study, such as sotalol, quinidine, amiodarone, erythromycin, clozapine, and clomipramine[Detail of drugs is documented in “Concomitant medication(s)”]; (j) resistance to conventional drugs. (Treatment resistance is defined as the failure to experience a therapeutic response during acute exacerbation of schizophrenia after adequate treatment with marketed antipsychotic agents on two or more occasions based on the judgment of the investigator during the 2 years before study entry); (k) known allergy to ziprasidone; (l) use of long-acting antipsychotics in the previous one month before the screening; or (m) participation in other studies within the last 30 days before the current study began and/or while participating in this research.

### Dosage and drug administration—ziprasidone IM

The initial dose of ziprasidone IM was between 10 to 20 mg. To achieve better clinical efficacy, most patients are recommended to start with 20 mg. Some patients who are thin or very sensitive to the drug are recommended 10 mg. The proposal is for reference only, as doctors should determine the starting dose according to the medical history of the patients.

The maximum dose was 40 mg/day. Doses of 10 mg may be administered every 2 h; doses of 20 mg may be administered every 4 h, up to a maximum of 40 mg/day.

Ziprasidone was injected intramuscularly as a sterile lyophilized powder, packaged in clear glass vials with fluted stoppers, and an aluminum shell containing 30 mg of ziprasidone mesylate. This mixture was reconstituted with sterile water for injection. The resulting solution contained 20 mg/mL and was administered intramuscularly.

### Oral ziprasidone

After IM treatment, the initial dose of oral ziprasidone was in the range of 40–80 mg and could be taken during dinner (or lunch) on the day of the last injection. Subjects then received a flexible dosage of oral ziprasidone, 120–160 mg/kg/day (i.e., twice daily with food), depending on the clinical response until day seven. Related studies have found that the peak plasma concentration of a 20 mg IM ziprasidone injection was similar to that achieved after oral ingestion of 80 mg twice every day [27], which can be used as a reference for sequential initial dose. However, doctors should judge the initial oral dose and the target dose according to the patient’s medical history and drug response. If the patient cannot tolerate the drug, the dose can be reduced at any time.

### Concomitant medication(s)

The concomitant use of drugs that may induce QTc prolongation during the study is prohibited, including antiarrhythmic agents (i.e., quinidine, procainamide, disopyramide, amiodarone, sotalol, bretylium, prenylamine, lidoflazine, terodiline) and others (i.e., arsenic trioxide, halofantrine, levomethadyl acetate, mesoridazine, thioridazine, pimozide, sparfloxacin, gatifloxacin, moxifloxacin, dolasetron mesylate, mefloquine, sertindole, or cisapride).

During the study, for subjects using concomitant medications, such as diuretics, the electrolyte levels, including serum potassium and magnesium, should be monitored. Patients with low serum potassium and/or magnesium levels should be treated with these electrolytes.

Administration of other antipsychotic agents and parenteral benzodiazepines, including IM and oral formulations, was not permitted during the study.

Oral benzodiazepines and other sedative and hypnotic drugs may be administered to treat insomnia pro re nata per night.

Further, benztropine and propranolol may be used during the drug treatment period to remedy EPS, but not as a prophylactic measure.

If any of these drugs are used by a subject during the study due to treatment requirements for their condition, the use of these drugs should be permitted and the study terminated.

### Overview of the study design

Altogether, six visits were predetermined for a typical sequential treatment: Visit 1 (baseline), Visit 2 (4 h), Visit 3 (1^st^ day), Visit 4 (3^rd^ day), Visit 5 (4^th^ day), and Visit 6 (7^th^ day). The vital signs were recorded during Visits 1, 3, 4, 5, and 6. PANSS was assessed at Visits 1, 3, 4, and 6. Furthermore, Clinical Global Impression of Severity (CGI-S) [28] was assessed at Visit 1, 3, 4, 5, and 6, but the Behavioral Activity Rating Scale (BARS) [29] score was examined at each visit. The Barnes akathisia scale and ECG were assessed at Visit 1, 4, and 6. Throughout the study, the prescribed dosage, concomitant medications, and adverse events were recorded. The effects of extrapyramidal syndrome and changes in ECG about ziprasidone were investigated.

### Efficacy evaluation criteria

The change in PANSS total scores from baseline to the endpoint was regarded as the primary efficacy endpoint.

Secondary efficacy endpoints included the change in PANSS-EC scores from the baseline to the end of the 1^st^ course of injection; the change from baseline in PANSS total and its subscales and PANSS-EC score over time through treatment; the change in PANSS score of early psychosis factors (i.e., conceptual disorganization, hallucinatory behavior, unusual thought content) from the Visit 1 to Visit 3, Visit 4, and Visit 6; and the change from baseline in BARS and CGI-S scores over time.

### Safety evaluations

We report adverse events (AEs) that occurred during the study period and verify the accuracy of the clinical report based on the relevant scale assessment and physical test indicators. While evaluating the side effects of EPS on the clinical report and the positive values of the Barnes scale, doctors often recommend drugs like diphenhydramine hydrochloride. Accordingly, we assessed the QTc interval level based on the ECG report, and sinus tachycardia was also reported in the ECG. Vital sign monitoring was used to assess whether patients have tachycardia, elevated blood pressure, and fever during the study period. Excessive sedation, insomnia, and dizziness were investigated through the clinical report concerning AEs. When the BARS indicator was < 3, we identified those subjects as patients with excessive sedation.

### Sample size

The primary efficacy variable used for power calculation was the PANSS total score reduction after seven days from baseline. According to the previous literature [30], the PANSS scores dropped by 15 with a standard deviation of 12.1 after 7 days. With the boundary at 12 points, *α* = 0.05, and *β* = 0.25, we estimated that we needed at least 80 subjects using a two-tailed test. In consideration of the rate of withdrawal, the sample size was calculated to be 95.

### Statistical methods

According to the intention-to-treat principle, the lack of primary efficacy outcomes gets carried forward with the previous results. Missing values of comparability analysis and secondary efficacy outcomes should not be carried forward, and analysis should be carried out with complete data. Categorical variables mainly focus on the comparative analysis of north China and northeast China. Descriptive statistical analyses were performed for all variables, including demographic variables, baseline, scores of each scale, safety variables, and so on. For quantitative data, including PANSS/PANSS-EC/CGI/BARS scores, mean, standard deviation, and range from maximum to minimum, repeated measures ANOVA was used to assess whether the dynamics of the above values are significantly different, and *p*-value < 0.05 was considered statistically significant. Statistics on the frequency of AEs in this experiment were provided for safety evaluation.

## Results

### Patient population

**Fig. 1:**
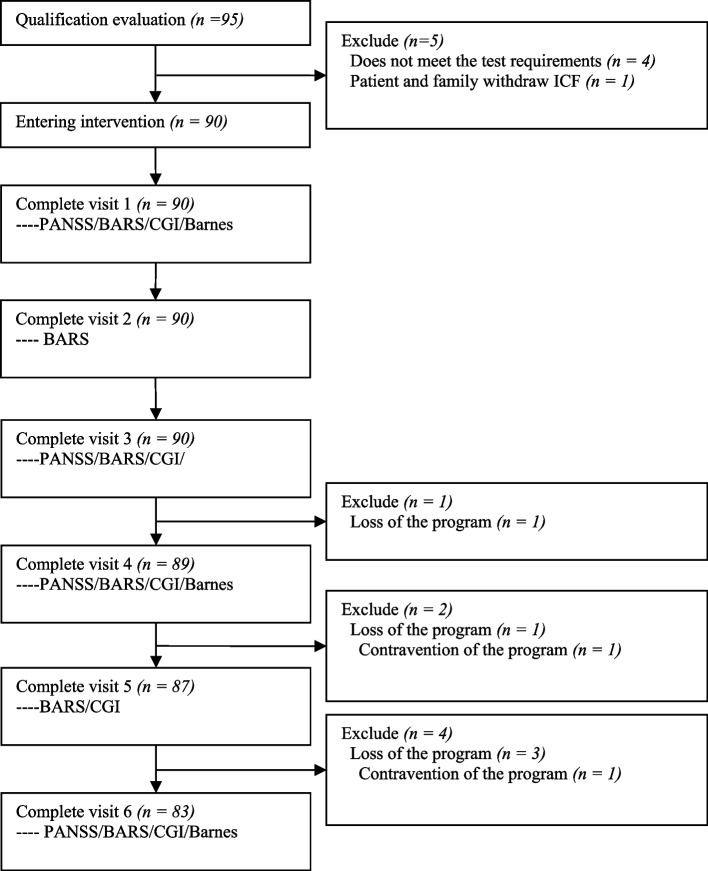
Flow chart of the study

A total of 95 randomized subjects were enrolled from three centers, among which 83 were tested and entered the safety and efficacy analysis. Twelve patients dropped out of the trial after signing the informed consent. The reasons for prematurely ​terminating the trial include factors such as the physician's assessment did not meet the test requirements (*n* = 4; 33.3%), withdrawal of the informed consent form by the patient and the family (*n* = 1; 8.33%), the loss of the program (*n* = 5; 41.6%), the contravention of the program (e.g., the medication and treatment of the contravention program) (*n* = 2; 16.7%). The final data efficiency was 87.3% (Fig. 1). Table 1 summarizes the demographic characteristics of the subjects who completed the trial at baseline. Notably, one of our three sub-centers was located in northeastern China, and the remaining were situated in northern China. The numbers of subjects collected for analysis in these two regions were 55 and 28.

**Table 1:** Baseline characteristics of patients enrolled in the study (*n* = 83)

**Age (years)**	32.06 ± 10.06 (18 – 59)
**Gender (**Male: Female**)**	41:42
**Weight, kg**	
Mean ± SD	67.81 ± 13.31
Range	45 – 103
**Height, cm**	
Mean ± SD	168.59 ± 6.84
Range	157 – 183
**BMI**	
Mean ± SD	23.68 ± 3.84
Range	15.90 – 37.11
**Duration of schizophrenia, years**	
Mean ± SD	5.83 ± 5.59
Range	0 – 29
**PANSS**	
Mean ± SD	90.87 ± 15.66
Range	51 – 125
**Positive PANSS subscale**	
Mean ± SD	26.78 ± 4.35
Range	16 – 37
**Negative PANSS subscale**	
Mean ± SD	19.07 ± 8.13
Range	7 – 38
**General psychopathology subscale**	
Mean ± SD	45.01 ± 7.71
Range	21 – 62
**PANSS-EC**	
Mean ± SD	19.52 ± 4.26
Range	8 – 32
**CGI**	
Mean ± SD	5.55 ± 0.70
Range	4 – 7
**BARS**	
Mean ± SD	5.82 ± 0.83
Range	5 – 7
**Heart rate (HR)**	
Mean ± SD	84.53 ± 11.95
Range	68 – 130
**Systolic blood pressure, mmHg**	
Mean ± SD	121.24 ± 13.81
Range	100 – 156
**Diastolic blood pressure, mmHg**	
Mean ± SD	77.49 ± 8.79
Range	56 – 96
**Temperature, ℃**	
Mean ± SD	36.47 ± 0.23
Range	36 – 37.2
**QTc interval of ECG**	
Mean ± SD	376.18 ± 41.17
Range	279 – 449

*BMI* Body Mass Index, *PANSS* Positive and Negative Syndrome Scale, *PANSS-EC* Excited Component of PANSS, *CGI* the Clinical Global Impressions scale, *BARS* the Behavioral Activity Rating Scale

### Clinical treatment

The doctor in charge administered 10–40 mg/d ziprasidone mesylate injection according to the patient's condition. Among them, 5 patients (6.0%) received 10 mg/d intramuscularly, 64 patients (77.1%) received 20 mg/d, 3 patients (3.6%) received 30 mg/d, and 11 patients (13.3%) received 40 mg/d. The average dosage was 22.41 ± 7.59 mg/d.

Doctors chose the sequential interval as within 2 h in 66 patients (79.5%), between 2 and 6 h in 13 patients (15.7%), and more than 6 h in 4 patients (4.8%).

**Fig. 2:**
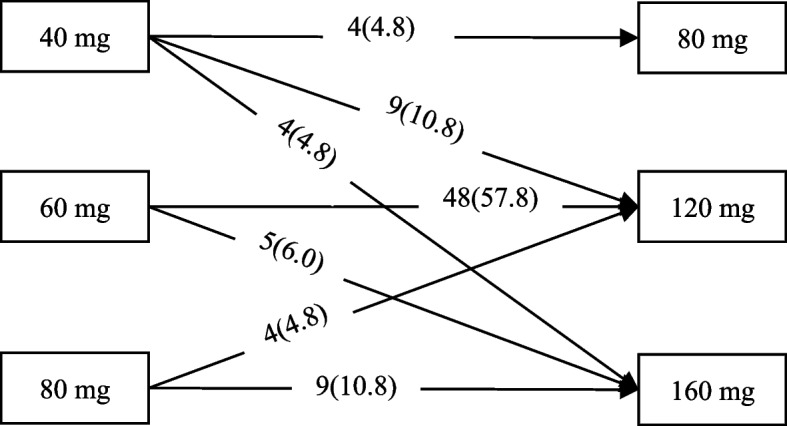
Selection from the first oral dose to the maximum oral dose [*n* (*%*)]

In terms of oral dose selection, the doctor in charge chose the first oral dose of 40–80 mg, with an average of 59.13 ± 12.06 mg; the maximum daily dose of ziprasidone was 80–160 mg/d, with an average of 126.75 ± 19.58 mg. The adjustment process from the first dose to the maximum dose is shown in Fig. 2.

### PANSS score

We observed that the PANSS scores at Visits 3, 4, and 6; the PANSS-positive, negative, general psychopathological subscale; and the PANSS-EC subscale demonstrated a decreasing trend, which was statistically significant (Table 2).

**Table 2:** Changes in the PANSS and its subscales and PANSS-EC scores vs. baseline values

	**Visit 1**	**Visit 3**	**Visit 4**	**Visit 6**	***F***	***P***
PANSS Total (*n* = *83*)	90.87 ± 15.66	80.87 ± 17.64	73.76 ± 18.10	64.93 ± 18.18	135.59	< 0.001
PANSS Total in North China (*n* = *55*)	93.55 ± 15.66	84.96 ± 16.34	78.74 ± 16.20	70.32 ± 16.24	89.50	< 0.001
PANSS Total in Northeast China (*n* = *28*)	85.61 ± 14.52	72.36 ± 17.48	62.30 ± 17.31	50.13 ± 15.00	57.72	< 0.001
PANSS Positive subscale (*n* = *83*)	26.78 ± 4.35	23.24 ± 5.04	20.61 ± 5.47	17.08 ± 4.84	145.30	< 0.001
PANSS Positive subscale in North China (*n* = *55*)	27.04 ± 4.30	24.31 ± 4.80	22.11 ± 5.28	18.42 ± 4.22	97.02	< 0.001
PANSS Positive subscale in Northeast China (*n* = *28*)	26.29 ± 4.49	21.07 ± 4.90	17.50 ± 4.52	13.72 ± 4.76	61.83	< 0.001
PANSS Negative subscale (*n* = *83*)	19.07 ± 8.13	18.04 ± 7.90	16.60 ± 7.83	16.20 ± 7.98	34.56	< 0.001
PANSS Negative subscale in North China (*n* = *55*)	21.16 ± 8.33	20.13 ± 8.15	18.83 ± 8.10	18.20 ± 8.05	25.14	< 0.001
PANSS Negative subscale in Northeast China (*n* = *28*)	14.96 ± 5.97	13.93 ± 5.50	12.22 ± 5.00	11.11 ± 5.16	9.16	< 0.001
General psychopathology subscale (*n* = *83*)	45.01 ± 7.71	39.70 ± 8.83	36.46 ± 8.58	32.11 ± 8.37	115.23	< 0.001
General psychopathology subscale in North China (*n* = *55*)	45.35 ± 7.82	40.79 ± 8.03	38.13 ± 7.80	34.27 ± 7.89	83.33	< 0.001
General psychopathology subscale in Northeast China (*n* = *28*)	44.36 ± 7.60	37.44 ± 10.10	32.84 ± 9.21	26.41 ± 6.94	39.25	< 0.001
PANSS-EC subscale (*n* = *83*)	19.52 ± 4.26	15.11 ± 4.39	12.51 ± 4.18	9.45 ± 3.57	157.12	< 0.001
PANSS-EC subscale in North China (*n* = *55*)	19.40 ± 3.90	15.83 ± 3.83	13.41 ± 4.07	10.07 ± 3.67	107.79	< 0.001
PANSS-EC subscale in Northeast China (*n* = *28*)	19.75 ± 4.96	13.67 ± 5.10	10.65 ± 3.84	7.89 ± 2.83	55.93	< 0.001

*PANSS* Positive and Negative Syndrome Scale, *PANSS-EC* Excited Component of PANSS

We also determined from the PANSS indicators that the effect in Northeast China was better than that in North China. There was a significant difference in the interventions between the two regions (Table 2).

### BARS & CGI-S score

We also observed the aforementioned subtraction trend on the BARS and CGI-S scores, as they significantly decreased during the study period and had a significant gap at the end of the study compared to the baseline.

The percentage of BARS ≤ 4 points (i.e., estimated to be completely unagitated) at the six access points, from Visit 1 to Visit 6, was taken as the percentage of the total sample, and it was found that BARS had a significant reduction at Visit 2 (i.e., 4 h of intramuscular injection). Over time, the proportion of subjects who completed sequential therapy gradually increased, and eventually, 92.8% of the subjects who were able to complete the sequential trial had a BARS level of up to 4 points (Visit 2: *n* = 36, 43.4%; Visit 3: *n* = 40, 48.2%; Visit 4: *n* = 59, 71.1%; Visit 5: *n* = 70, 84.3%; Visit 6: *n* = 77, 92.8%). CGI during Visit 1 and Visit 3–Visit 6 can also be elicited to acquire 4 points (i.e., a moderate level of mental illness), and the following levels were completed for 7.2%, 26.5%, 46.9%, 53.2%, and 67.7% of the total sample, respectively. Additionally, we also found that in the BARS score, the effectiveness of the northeast seems to be better than that of northern China. This outcome was consistent with the PANSS results (Table 3).

**Table 3:** Number and percentage of BARS ≤ 4 points

	Visit 1	Visit 2	Visit 3	Visit 4	Visit 5	**Visit 6**
BARS ≤ 4 (Total), *n*(*%*)	0(0)	36(43.4)	40(48.2)	59(71.1)	70(84.3)	77(92.8)
BARS ≤ 4 (North China), *n* (*%*)	0(0)	22(40.0)	25(45.5)	34(61.8)	44(80.0)	50(90.9)
BARS ≤ 4 (Northeast China), *n* (*%*)	0(0)	14(50.0)	15(53.6)	25(89.3)	26(92.9)	27(96.4)

*BARS* the Behavioral Activity Rating Scale

### Adverse reaction

During the entire experimental period, a total of 62 AEs occurred, which may or may not be associated with the drugs (Table 4).

**Table 4:** Listing of 62 Adverse events

	**Number of reports (*n*)**	**Percentage (*%*)**
Extrapyramidal symptoms (Grade 1, Slightly without drug intervention)	10	12.0
Extrapyramidal symptoms (Grade 2–3, Serious need for drug intervention)	13	15.7
Prolonged QTc interval (≥ 450 ms)	7	8.4
Prolonged QTc interval (> 60 ms change from baseline)	6	7.2
Sinus tachycardia	2	2.4
Sinus bradycardia	1	1.2
Hypertension	6	7.2
Excessive sedation	10	12.0
Insomnia	6	7.2
Dizziness	1	1.2

*QTc* the heart rate-corrected QT

In the study period, the QTc interval in 7 cases extended 450 ms at either Visit 4 or 6, the QTc intervals in 6 cases were greater than 60 ms from the baseline (Table 4). Nonetheless, the clinicians continued to complete the study after the assessment, and at the end of the study, the QTc interval returned to the normal range. We retested these subjects 1 month later, and the QTc interval means ± SD was 365.10 ± 25.33 ms. We found that intramuscular ziprasidone dose was associated with a trend in QTc interval prolongation in V4-V1 (Spearman's correlation coefficient 0.716, *P* = 0.006), but the oral dose was not associated with a trend in QTc interval prolongation in V6-V1 (Spearman correlation coefficient—0.288, *P* = 0.339) (Table 5).

**Table 5:** The QTc interval extended during the trial

	Visit 1 (HR)	Visit 4 (HR)	**Visit 6** (HR)	Visit 4 vs Visit 1	**Visit 6** vs Visit 1	Intramuscular dose	oral dose	QTc after 1 month (HR)
**QTc interval ≥ 450 ms during the study period**	326(80)	450(80)	430(88)	124	104	30	160	398(76)
	410(71)	450(70)	445(72)	40	35	20	160	312(68)
	396(82)	457(76)	437(76)	61	41	20	120	348(70)
	406(76)	450(82)	459(88)	44	53	20	120	426(74)
	446(84)	451(84)	453(80)	61	41	20	160	368(75)
	402(78)	441(75)	497(74)	39	95	20	80	426(76)
	390(78)	400(80)	500(70)	10	110	20	120	358(71)
**QTc interval was greater than the baseline level (≥ 60 ms) during the study period**	359(92)	440(80)	443(80)	81	84	40	120	368(88)
	310(78)	340(80)	400(80)	30	90	20	80	384(78)
	314(84)	357(84)	381(76)	43	67	20	80	338(80)
	348(95)	397(84)	425(96)	49	77	20	120	356(80)
	321(98)	403(96)	400(93)	82	79	30	120	356(93)
	325(85)	340(90)	415(78)	15	110	20	120	372(80)

*QTc* the heart rate-corrected QT

## Discussion

In Chinese psychiatric hospital wards, the control of agitation symptoms follows the principles of comprehensive treatment and adopts a variety of preventive measures. There are two commonly used clinical methods to control agitation. The first method involves the use of antipsychotic drugs, which work by inhibiting the dopaminergic neuron hyperfunction. The second method utilizes benzodiazepine drugs, which enhance the inhibition of γ-aminobutyric acid (GABA) neurons to produce sedation, but fail to control psychosis symptoms [12]. Electroconvulsive therapy (ECT) is a method that has a longer history than psychiatric drugs and is more reliable in controlling agitation. It is still widely used in Chinese psychiatric hospitals. However, in some developing countries, the use of anesthesia and succinylcholine has not been modified before ECT [31]. The BETA guidelines advocate the early application of verbal de-escalation techniques. In China, although healthcare workers have not received systematic training in this technology, everyone knows to control the agitation of patients by pacificate them.

In recent years, many studies have been conducted with haloperidol or placebo as a control to assess the efficacy and safety of new antipsychotics in controlling schizophrenia. In 2005, a double-blind, randomized controlled study of schizophrenia symptoms was conducted using ziprasidone and haloperidol injections. The findings signified that ziprasidone was better than haloperidol in the reduction of BARS. Meanwhile, ziprasidone had a lower incidence of EPS than haloperidol [22].

Similarly, the sequential therapy of ziprasidone, a transition from IM to oral, was studied in Italy via multiple centers. A total of 150 patients were enrolled, and a decrease in PANSS and CGI-S scores was observed throughout the study. These reductions were significant compared to the transition from IM to oral preparations [19].

This study also used sequential therapy in the first 3 days to take full advantage of the rapid onset of ziprasidone injection, and oral dosage forms were targeted in the last 4 days. This contributes to the treatment in terms of stability and continuity before and after the use of the same drug. After evaluation, it was found that PANSS score, PANSS-EC, and BARS score showed a progressive decreasing trend after treatment, with statistical significance (*P* < 0.001), suggesting that treatment has a significant effect. With the prolongation of treatment time, the control effect became more obvious. Particularly, the BARS score suggested that the ziprasidone injection enables rapid control of agitation. The order for the oral dosage form, although not as beneficial as the progress of injection, continues to affect and promote the stability of the role of the disease. While controlling agitation, the use of such sequential treatment can effectively control agitation among 92.8% of the patients.

This multicenter study involved three selected centers located in North China (i.e., near Beijing) and Northeast China (i.e., near Harbin). We found some differences across the two regions, even though the symptoms of both groups were controlled accordingly. The effect of control in the Northeast China region seems to be better than that of subjects in North China region. This finding may be related to the characteristics of subjects in different regions, local cultural differences, ward management, and other factors.

The adverse reaction highlighted in this study mainly correlated with extrapyramidal syndrome (27.7%). This ratio is higher than that observed in previous studies; however, we also found that most patients’ EPS symptoms were mild and could be tolerated without drugs, while only 15.7% required anticholinergic drugs to eliminate this side effect.

Traditionally, ziprasidone led to prolonged QTc prolongation, but this study found that 7-day sequential treatment of ziprasidone did not lead to a severe prolonged QTc interval. Among these, the QTc interval extended beyond the normal range in only seven cases, with an incidence of 8.4%, but with individual-level differences. Furthermore, in an additional follow-up for QTc prolongation, 1 month after the study, we found that the prolongation of the QTc interval returned to shortening. However, we found that QTc prolongation in the first 3 days was related to the dose of ziprasidone injection. There are other cases of research finding similar phenomena [31]. However, if ziprasidone injection was discontinued, the QTc prolongation recovered somewhat [32].

Nonetheless, some studies suggest that haloperidol leads to an extended QTc interval of 23% [33]. There are also related studies, which outline that the QTc interval prolongation of ziprasidone is not more serious than that of haloperidol [16, 34]. Although this study has confirmed that the short-term use of ziprasidone has a lower risk to the heart, we recommend that electrocardiography be performed within 4 days of the initial use and end of the intramuscular injection to ensure the safety of the treatment.

Despite our efforts, this study had three shortcomings. First, no control group can help accurately check the difference in the efficacy and side effects of sequential therapy with new atypical antipsychotics. The relatively small sample size also limits the elucidation of the difference. Second, the observation period of the experiment was only 7 days, and no long-term follow-up was performed to evaluate whether ziprasidone could safely improve the patients’ psychiatric symptoms as a first-line drug after successful sequential therapy. Third, although the regional differences in efficacy were considered in our study by evaluating centers in Northeast and North China regions, the current research data are not enough to support a definite conclusion. Furthermore, these centers did not include the southern and western China regions, where the 2017 Chinese agitation survey showed that residence is a risk factor for agitation in newly hospitalized schizophrenia patients [6]. In future research, we shall consider expanding the study to more regions and making further comparisons of effects.

In summary, in the Chinese psychiatric ward, the use of ziprasidone is relatively uncommon, and many clinical psychiatrists have a limited understanding of this drug. In this study, ziprasidone was presented sequentially to treat acute schizophrenia, providing a new alternative to physicians working in a psychiatric emergency environment.

## Conclusions

A ziprasidone injection can lead to rapid control over agitated patients, and its sequential oral dosage form can ensure further efficacy. Even though different regions specify some differences, the most common adverse reaction instigated by ziprasidone is extrapyramidal syndrome and excessive sedation. QT prolongation did occur during treatment of acute agitation, but it was rare and was found to be reversible at the 1-month follow-up. Other serious injuries to the body are considered less common. Overall, the safety profile of ziprasidone is reliable.

## Data Availability

The datasets used and analyzed during the current study are available from the corresponding author upon reasonable request.

## Abbreviations

5-HT: 5-Hydroxytryptamine/serotonin
AEs: Adverse events
BARS: The Behavioral Activity Rating Scale
BETA: The best practices in the evaluation and treatment of agitation
BMI: Body Mass Index
CGI: The Clinical Global Impressions scale
CGI-I: The Clinical Global Impressions-Improvement scale
CGI-S: The Clinical Global Impressions-Severity of Illness
D: Dopamine
DSM-IV: The Diagnostic and Statistical Manual of Mental Disorders Fourth Edition
ECG: Electrocardiogram
ECT: Electroconvulsive therapy
EPS: Extrapyramidal symptoms
GABA: γ-Aminobutyric acid
IM: Intramuscular
PANSS: The Positive and Negative Syndrome Scale
PANSS-EC: PANSS-excited component
QTc: The heart rate-corrected QT
ZIMO: Ziprasidone Versus Haloperidol in Sequential IM/Oral Treatment
